# Wearable Data From Subjects Playing Super Mario, Taking University Exams, or Performing Physical Exercise Help Detect Acute Mood Disorder Episodes via Self-Supervised Learning: Prospective, Exploratory, Observational Study

**DOI:** 10.2196/55094

**Published:** 2024-07-17

**Authors:** Filippo Corponi, Bryan M Li, Gerard Anmella, Clàudia Valenzuela-Pascual, Ariadna Mas, Isabella Pacchiarotti, Marc Valentí, Iria Grande, Antoni Benabarre, Marina Garriga, Eduard Vieta, Allan H Young, Stephen M Lawrie, Heather C Whalley, Diego Hidalgo-Mazzei, Antonio Vergari

**Affiliations:** 1 School of Informatics University of Edinburgh Edinburgh United Kingdom; 2 The Alan Turing Institute London United Kingdom; 3 Bipolar and Depressive Disorders Unit Department of Psychiatry and Psychology Hospital Clínic de Barcelona Barcelona Spain; 4 Institut d’Investigacions Biomèdiques August Pi i Sunyer Barcelona Spain; 5 Centro de Investigación Biomédica en Red de Salud Mental Instituto de Salud Carlos III Madrid Spain; 6 Departament de Medicina Facultat de Medicina i Ciències de la Salut Universitat de Barcelona Barcelona Spain; 7 Institute of Psychiatry, Psychology and Neuroscience King's College London London United Kingdom; 8 Division of Psychiatry Centre for Clinical Brain Sciences University of Edinburgh Edinburgh United Kingdom; 9 Generation Scotland Institute for Genetics and Cancer University of Edinburgh Edinburgh United Kingdom

**Keywords:** mood disorder, time-series classification, wearable, personal sensing, deep learning, self-supervised learning, transformer

## Abstract

**Background:**

Personal sensing, leveraging data passively and near-continuously collected with wearables from patients in their ecological environment, is a promising paradigm to monitor mood disorders (MDs), a major determinant of the worldwide disease burden. However, collecting and annotating wearable data is resource intensive. Studies of this kind can thus typically afford to recruit only a few dozen patients. This constitutes one of the major obstacles to applying modern supervised machine learning techniques to MD detection.

**Objective:**

In this paper, we overcame this data bottleneck and advanced the detection of acute MD episodes from wearables’ data on the back of recent advances in self-supervised learning (SSL). This approach leverages unlabeled data to learn representations during pretraining, subsequently exploited for a supervised task.

**Methods:**

We collected open access data sets recording with the Empatica E4 wristband spanning different, unrelated to MD monitoring, personal sensing tasks—from emotion recognition in Super Mario players to stress detection in undergraduates—and devised a preprocessing pipeline performing on-/off-body detection, sleep/wake detection, segmentation, and (optionally) feature extraction. With 161 E4-recorded subjects, we introduced E4SelfLearning, the largest-to-date open access collection, and its preprocessing pipeline. We developed a novel E4-tailored transformer (E4mer) architecture, serving as the blueprint for both SSL and fully supervised learning; we assessed whether and under which conditions self-supervised pretraining led to an improvement over fully supervised baselines (ie, the fully supervised E4mer and pre–deep learning algorithms) in detecting acute MD episodes from recording segments taken in 64 (n=32, 50%, acute, n=32, 50%, stable) patients.

**Results:**

SSL significantly outperformed fully supervised pipelines using either our novel E4mer or extreme gradient boosting (XGBoost): n=3353 (81.23%) against n=3110 (75.35%; E4mer) and n=2973 (72.02%; XGBoost) correctly classified recording segments from a total of 4128 segments. SSL performance was strongly associated with the specific surrogate task used for pretraining, as well as with unlabeled data availability.

**Conclusions:**

We showed that SSL, a paradigm where a model is pretrained on unlabeled data with no need for human annotations before deployment on the supervised target task of interest, helps overcome the annotation bottleneck; the choice of the pretraining surrogate task and the size of unlabeled data for pretraining are key determinants of SSL success. We introduced E4mer, which can be used for SSL, and shared the E4SelfLearning collection, along with its preprocessing pipeline, which can foster and expedite future research into SSL for personal sensing.

## Introduction

Mood disorders (MDs) are a group of mental health conditions in the *Diagnostic and Statistical Manual, Fifth Edition* (DSM-5) classification system [1]. They are chronic, recurrent disorders featuring disturbances in emotions, energy, and thought, standing out as a leading cause of worldwide disability [2,3] and suicidality [4]. Timely recognition of MD episodes is critical toward better outcomes [5]. However, this is challenging due to generally limited patient insight [6], compounded with the low availability of specialized care for MDs, with rising demand straining current capacity [7,8].

Personal sensing, involving the use of machine learning (ML) to harness data passively and near-continuously collected with wearable devices from patients in their ecological environment, has been attracting interest as a promising paradigm to address this gap [9]. Indeed, some of the core MD clinical features (eg, disturbance in mood and energy levels) translate into changes in physiological parameters measurable with wearable devices [10-12]. A major barrier to the development of clinical decision support systems featuring personal sensing has been the scarcity of labeled data, that is, data with annotations by clinicians about the MD state (eg, diagnosis, disease phase, symptom severity). Collecting and annotating data for personal sensing in MDs is, indeed, an expensive and time-consuming enterprise; thus, studies typically use samples running into only a few dozen patients [13-20].

**Figure 1:**
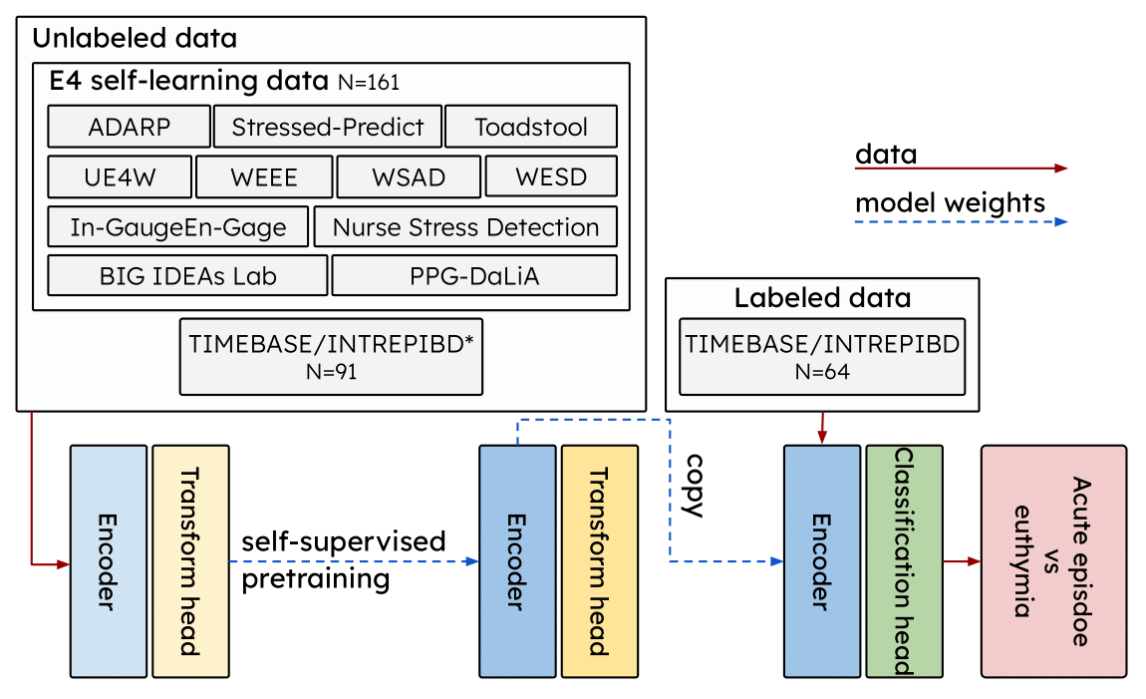
A total of ∼6254 hours (261 days) of unlabeled recordings from 252 subjects while awake were used for self-supervised pretraining. Unlabeled data comprised a collection of 11 open access data sets, whose aggregation we make publicly available (E4SelfLearning), along with part of the TIMEBASE/INTREPIBD study that was not relevant for the target task under investigation (ie, acute episode vs euthymia classification). Unlabeled data were passed through a model consisting of an encoder and a transform head for self-supervised pretraining; the pretrained encoder block was then retained for the target task, while the transform head was replaced with a new, randomly initialized classification head. ∗The target task (labeled) training set from the TIMEBASE/INTREPIBD study was also used during self-supervised pretraining. Further details on the data sets used in this study are available in Table S1 in Multimedia Appendix 1. ADARP: Alcohol and Drug Abuse Research Program; PGG-DaLia: PPG Dataset for Motion Compensation and Heart Rate Estimation in Daily Life Activities; TIMEBASE/INTREPIBD: Identifying Digital Biomarkers of Illness Activity in Bipolar Disorder/Identifying Digital Biomarkers of Illness Activity and Treatment Response in Bipolar Disorder; UE4W: Unlabeled Empatica E4 Wristband; WEEE: Wearable Human Energy Expenditure Estimation; WESAD: Wearable Stress and Affect Detection; WESD: Wearable Exam Stress Dataset.

In this work, we took a different perspective and leveraged *unlabeled* data collected with the Empatica E4 (hereafter E4) wristband [21], a popular research-grade device for personal sensing studies [22], as well as recent advancements in self-supervised learning (SSL) techniques that can learn meaningful representations from such unlabeled data. Specifically, we took advantage of open access data sets that record physiological data with the E4 across different settings but do not address MDs and therefore do not provide information about the mood state of the subjects involved. Although each such data set has only a limited number of subjects, our aggregated and preprocessed data set E4SelfLearning can break the labeled data bottleneck for personal sensing in MDs (Figure 1) [23-33].

Fully supervised systems require vast amounts of data to train, thus limiting their application in different fields, such as health care, where amassing large, high-quality data sets is demanding in terms of time and human resources [34]. Although previous studies on personal sensing for MDs have investigated different tasks, including acute MD episode detection [13-16], regression of a psychometric scale total score [17-19], and, more recently, multitask inference of all items in 2 commonly used psychometric scales [35], they all developed their models in a fully supervised fashion (ie, they were trained on samples for which ground-truth labels were available). As a result, considering that obtaining clinical annotations from patients, especially when on an acute MD episode, is a challenging and expensive enterprise, the sample size is generally modest (eg, N=52 in Côté-Allard et al [15], N=45 in Tazawa et al [13], and N=31 in Pedrelli et al [18]).

SSL, in contrast, is a framework where the model creates proxy supervisory signals within the data themselves, therefore alleviating the annotation bottleneck and allowing us to repurpose existing unlabeled data sets [36]. Specifically, SSL derives supervisory signals from the data themselves, thanks to pretext tasks, which are new supervised challenges, for example, imputing occluded parts of the input data. Through such preparatory pretext tasks, not requiring expert annotation, the model learns useful representations, partial solutions to the downstream target task of interest, for which only a comparatively small amount of annotated data are available [37]. On the back of the great success of SSL in computer vision (CV) [37] and natural language processing (NLP) [38], and with encouraging findings in other health care applications [39], we extended pioneering SSL works on multivariate time series [40-42] to personal sensing in MDs.

**Figure 2:**
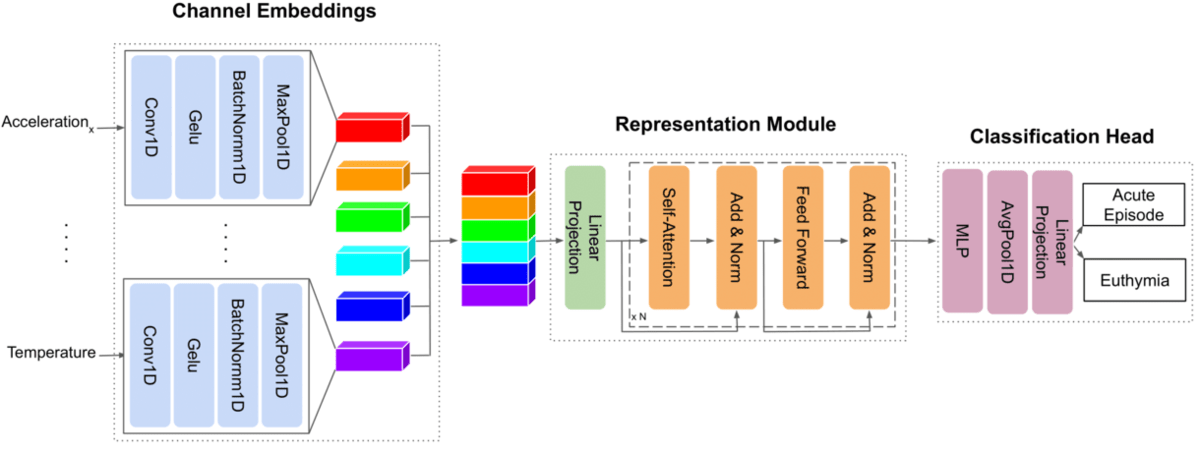
E4mer is a transformer model tailored to the Empatica E4 input data. E4mer consists of 3 sequential modules: (1) channel embeddings set in parallel, 1 for each Empatica E4 raw input channel (ie, acceleration_x_, acceleration_y_, acceleration_z_, BVP, EDA, TEMP), extracting features and mapping channels to tensors of dimensionality (B=batch size, N=time steps, F=number of filters) so that they can be conveniently concatenated along dimension F; (2) RM learning contextual representations of the input time steps within the input segment, thanks to the multihead self-attention mechanism; (3) classification head outputting probabilities for the 2 target classes (ie, acute MD episode and euthymia). SSL models used in our experiments featured the same E4mer architecture described before, where, however, the classification head was replaced with a transform head projecting onto a label space compatible with the pretext task at hand. BVP: blood volume pressure; E4mer: E4-tailored transformer; EDA: electrodermal activity; MD: mood disorder; MLP: multilayer perceptron; RM: representation module; SSL: self-supervised learning; TEMP: temperature.

In this work, we made the following contributions:

- We gathered 11 open access data sets recording physiological data with an E4 wristband and developed a pipeline for preprocessing such data that performed on-/off-body detection, sleep/wake detection, segmentation, and (optionally) feature extraction. We made the preprocessing pipeline and the preprocessed data publicly available. This collection (E4SelfLearning), with 161 subjects, is the biggest open access data set to date. We believe that this effort can stimulate future research into SSL with multivariate time-series sensory data by removing 2 barriers, preprocessing and data availability.
- We proposed a novel E4-tailored transformer (E4mer) architecture (Figure 2) [43] and showed that SSL is a viable paradigm, outperforming both fully supervised E4mer and classical machine learning (CML) models using handcrafted features in distinguishing acute MD episodes from clinical stability (euthymia in psychiatric parlance), that is, a time-series (binary) classification task.
- We investigated what makes SSL successful. Specifically, we compared 2 main pretext task designs (ie, masked prediction [MP] and transformation prediction [TP]) [44], and for the best-performing routine, we studied its sensitivity to the unlabeled data availability in ablation analyses. We inspected learned embeddings and showed that they capture meaningful semantics about the underlying context (ie, sleep/wake status) and symptom severity.

## Methods

### Study Sample

#### The TIMEBASE/INTREPIBD Cohort

Our target task was to distinguish acute MD episodes from euthymia using wearable data. We started from a data set for which we had labeled samples, the TIMEBASE/INTREPIBD (Identifying Digital Biomarkers of Illness Activity in Bipolar Disorder/Identifying Digital Biomarkers of Illness Activity and Treatment Response in Bipolar Disorder) cohort [45]. A detailed description of the data collection campaign was given by Anmella et al [45]. In brief, this was a prospective, exploratory, observational study conducted at the Hospital Clìnic, Barcelona, Spain. Patients with a DSM-5 diagnosis of either major depressive disorder (MDD) or bipolar disorder (BD) were enrolled either in the acute affective episode group (defined according to the “Structured Clinical Interview” for DSM-5 disorder criteria) or in the euthymia group (score≤7 on the Hamilton Depression Rating Scale-17 [46] and the Young Mania Rating Scale [47] for at least 8 weeks [48], as confirmed with weekly ambulatory assessments). The former group had post–acute-phase follow-ups, which were, however, excluded from all analyses presented here. At the time of conducting this study, a total of 64 patients were available for the target task, half in the acute affective episode group and half in the euthymia group. Additionally, an extra 91 subjects (including healthy controls, subjects with schizophrenia, and subjects with a substance abuse disorder), whose status was not relevant to the target task, were available from the TIMEBASE/INTREPIBD cohort for self-supervised pretraining.

Patients were interviewed by a psychiatrist collecting clinical demographics (Table 1 and Table S2 in Multimedia Appendix 1) and were required to wear on their nondominant wrist an E4 wristband until the battery ran out (~48 hours). The E4 records 3D acceleration (sampling rate 32 Hz), blood volume pressure (BVP, sampling rate 64 Hz), electrodermal activity (EDA, sampling rate 4 Hz), heart rate (HR, sampling rate 1 Hz), interbeat interval (IBI, ie, the time between 2 consecutive heart ventricular contractions), and skin temperature (TEMP, sampling rate 1 Hz).

As shown in Table 1, MD episodes clinically lie on a spectrum, with depression on one end and mania on the other; mixed episodes, featuring symptoms from both polarities, are a bridge between the 2 spectrum extremes. In this study, we considered acute MD episodes of any polarity, and similarly, we considered euthymia as a unique class, whether in the context of a BD or an MDD diagnosis. Medication classes administered to the cohort are shown in Table S2 in Multimedia Appendix 1; Bonferroni-corrected chi-square tests found no significant association between treatment status (being on a given drug class or not) and target class (acute affective episode vs euthymia).

**Table 1:** Clinical-demographic features of the target task (acute affective episode vs euthymia classification) population (N=64).

Features		Acute affective episode group (n=32)	Euthymia group (n=32)
Age (years), means (SD)		50.56 (13.05)	47.22 (16.06)
Females, n (%)		15 (46.9%)	14 (43.8%)
**MDE-BD^a^**			
	Patients, n (%)	9 (28.1)	—^b^
	HDRS^c^ score, mean (SD)	20.22 (6.34)	—
	YMRS^d^ score, mean (SD)	2.56 (3.94)	—
**MDE-MDD^e^**			
	Patients, n (%)	7 (21.9)	—
	HDRS score, mean (SD)	25.14 (4.78)	—
	YMRS score, mean (SD)	1.86 (2.41)	—
**ME^f^**			
	Patients, n (%)	14 (43.8)	—
	HDRS score, mean (SD)	5.67 (4.37)	—
	YMRS score, mean (SD)	20.13 (6.28)	—
**MX^g^**			
	Patients, n (%)	2 (6.2)	—
	HDRS score, mean (SD)	16 (4.24)	—
	YMRS score, mean (SD)	13.5 (4.95)	—
**BD^h^**			
	Patients, n (%)	—	26 (81.3)
	HDRS score, mean (SD)	—	2.93 (1.73)
	YMRS score, mean (SD)	—	1.3 (1.61)
**MDD^i^**			
	Patients, n (%)	—	6 (18.7)
	HDRS score, mean (SD)	—	3.14 (1.95)
	YMRS score, mean (SD)	—	0.29 (0.76)

^a^MDE-BD: major depressive episode in bipolar disorder.

^b^Not applicable.

^c^HDRS: Hamilton Depression Rating Scale-17.

^d^YMRS: Young Mania Rating Scale.

^e^MDE-MDD: major depressive episode in major depressive disorder.

^f^ME: manic episode.

^g^MX: mixed episode.

^h^BD: bipolar disorder.

^i^HDRS: Hamilton Depression Rating Scale-17.

#### E4SelfLearning

For self-supervised pretraining, we gathered 11 open access data sets recording with an E4 [23-33]. Although they all used the same hardware, software, and firmware, such data sets could differ substantially for population, recording setting, and task: from students taking exams [29] or attending classes [31] to nurses carrying out their duty [30] and subjects performing different physical activities [28] or playing Super Mario [27]. Subjects that were not part of the target classes from the TIMEBASE/INTREPIBD study were also included in the unlabeled data for SSL.

### Data Preprocessing

Our preprocessing encompassed the following sequential stages: on-/off-body detection, sleep/wake detection, segmentation, and (when preparing data for CML models) feature extraction.

During free-living wear, subjects might remove their device or contact with the wrist might be suboptimal. As a result, off-body periods can be erroneously mistaken for periods of sleep or sedentary behavior, due to the shared feature of an absence of movement. Signal discontinuity in biopotentials, such as EDA, due to a lack of skin contact can be reliably leveraged to detect nonwear periods. As shown by Vieluf et al [49] and Nasseri et al [50], we considered measurements less than 0.05 µS as indicative of off-body status. Furthermore, as we noticed occurrences of values greater than the EDA sensor range (ie, 100 µS [51]), as well as instances of TEMP values outside the physiological range (30°C-40°C), we set both to off-body.

As physiological data vary wildly across sleep and wake statuses, we used sleep/wake detection as a form of data cleaning to reduce the variance in the signal and considered only the wake time in our analyses, especially as most publicly available data sets are recorded in wake conditions. We opted for the algorithm developed by Van Hees et al (*Van Hees*) [52], which was reported as the best-performing algorithm in a recent benchmark study on sleep/wake detection (average *F*_1_-score=79.1) [53]. Like most nonproprietary algorithms, Van Hees uses triaxial acceleration and, specifically, relies on a simple heuristic defining sleep with the absence of a change in the arm angle >5° for 5 minutes or more. To accommodate this rule, wherever on-body sampling cycles did not constitute unbroken sequences of at least a 5-minute duration, all the measurements in that period were considered as off-body and discarded from further analysis.

The wake time from each recording was then segmented with a sliding window, whose segment length (ω) and step size (∆ω) were set to 512 and 128 seconds, respectively. This approach, also referred to as window slicing [54], is a common form of data augmentation in time-series classification as multiple segments are produced from a single recording, each one marked with the same label, and is common in personal sensing for MDs. Previous relevant works [15,18,55] have defined ω (∆ω) based on clinical intuition and convenience concerning the available data. Another work [35] investigating the regression of HDRS and the YMRS items found the optimal ω through tuning, a computationally expensive approach in our setting; however, it showed that ω was not among the most important hyperparameters for the task at hand. Here, we opted for 512 seconds (~8.5 minutes, conveniently a power of 2 for computational efficiency in binary computers), similar to the 5-minute intervals used by Panagiotou et al [55] for training neural autoencoder architectures on anomaly detection by reconstruction error estimation. Our choice was a trade-off between clinical insight and technical constraints. Clinical intuition suggests that too small a value of ω may be ill suited to capture enough information toward acute affective episode versus euthymia discrimination. However, unlabeled data sets used for self-supervised pretraining recorded relatively short sessions (eg 1 hour [26]). As both CML and deep learning models are trained on individual segments and too long a segment length equates to fewer training data points, a 512-second-long segment allowed us to have enough data for developing ML models [55].

Recording segments constituted our basic unit of analysis, and for the target task, segments from the same recording all shared the same ground-truth label (ie, either acute affective episode or euthymia). When fed to deep learning models, segments were channel-wise standardized by subtracting the mean and dividing by the SD. Such statistics were learned from the target task training set or, in the case of SSL, its aggregation with unlabeled data. Acceleration, the BVP, EDA, and TEMP were considered in deep learning models, while the HR and the IBI, as features derived from the BVP through a proprietary algorithm, were excluded from the deep learning experiments shown here (see Multimedia Appendix 1). However, when using CML, handcrafted features were extracted from segments using *FLIRT* [56], a popular open access feature extraction toolkit for the E4. Note that a single row of features per segment was extracted; in other words, the window size parameter in FLIRT was set equal to ω. We used all features available through this package, derived with the *flirt.acc.get_acc_features* (eg, acceleration entropy), *flirt.eda.get_eda_features* (eg, tonic and phasic EDA components), and *flirt.hrv.get_hrv_features* (eg, HR and HR variability measures) functions. As FLIRT does provide built-in functions for TEMP, we also extracted the segment mean (SD) for this channel. Any missing value was handled with mean imputation. The percentage rate of missing values had a range of 0-37.31, with a mean of 10.44 (SD 16.78).

### Data Splits and Metrics

In SSL experiments, we split unlabeled data in a ratio of 85:15 into train and validation sets, partitioning recordings across the 2 sets. For the target task, we investigated a time-split scenario, therefore splitting each recording into train, validation, and test sets again in a ratio of 70:15:15 along the recording time, thus testing generalization across future time points. We made sure that segments with overlapping motifs at the border between target task splits (resulting from using a sliding window with ∆ω<ω) were confined to 1 split only, thus ultimately producing 18896, 3904, and 4128 segments for the train, validation, and test sets. The target task validation set doubled as a test set for estimating generalization performance on the SSL pretext task. The time-split scenario is common in personal sensing for MDs (eg, [18,35]), and indeed, despite efforts toward learning subject-invariant representations [57,58], cross-subject generalization remains an unsolved challenge, so personal sensing systems typically require access to each subject’s physiological data distribution at training time [59].

The target task was a time-series binary classification. As expected in free-living wear, the total wear time and the off-body and wake times varies across subjects (and, as a result, so did the number of segments). Two-tailed *t* tests were performed to verify significant mean differences in off-body and wake times across individuals from the 2 target classes (acute affective episode and euthymia) but yielded a Bonferroni-corrected P value of >.05 (P=.56 for off-body time and P=.82 for wake time). An equal number of segments from each class was extracted for the target task. To that end, we found the pairing of euthymia and acute affective episode recordings that minimized the pairwise difference between the number of segments available per participant; next, within each pair, the first n segments were retained, where n is the number of segments of the shortest recording in the pair. We optimized models on the target task for segment-level accuracy (ACC_segment_). Second, to provide a subject-level perspective, we reported the subject ACC:



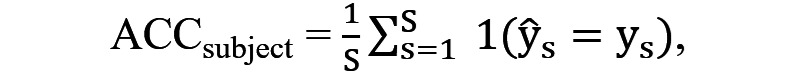



where y_s_ is the ground-truth mood state of the s-th subject, which is constant across all the s-th subject’s recording segments, and 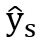 is a majority vote on the s-th subject, corresponding to the majority predicted class across the s-th subject’s recording segments.

### Machine Learning Models

We developed 2 types of baselines for the target task: (1) an E4-tailored deep learning pipeline inputting raw recording segments (E4mer) and (2) CML models using handcrafted features extracted with FLIRT from recording segments. We then assessed what boost in performance, if any, a self-supervised pretraining phase might deliver, where the SSL models shared the same building blocks as E4mer.

#### Baseline Models

##### E4-Tailored Transformer

E4mer is an artificial neural network discriminative classifier modeling the probability of an acute MD episode, given a recording segment. As shown in Figure 2, E4mer has 3 sequential blocks: (1) channel embeddings (CEs) set in parallel, consisting of the same 1D convolutions with a kernel size equal to the channel sampling frequency, followed by Gaussian error linear unit (Gelu) activation, 1D BatchNorm, and 1D MaxPooling using the channel sampling frequency as both kernel size and step size, so each CE output has the same dimensionality and can be conveniently concatenated with the others before being passed onto (2) a transformer [43] representation module (RM), and (3) a multilayer perceptron (MLP) classification head (H_sl_). The CEs extract features from the input E4 channels and are designed to handle channels sampled at different frequencies; the RM, powered by multihead self-attention, learns contextual representations of the input tokens (timestamps in our case) within a recording segment; lastly, the H_sl_ maps such representations onto a label space appropriate for a binary classification. E4mer was trained to minimize the binary cross-entropy (BCE) loss between acute affective episode/euthymia predictions and the corresponding ground truth.

##### Classical Machine Learning

We experimented with the following algorithms, given their popularity and state-of-the-art performance in biomedical applications [60], including personal sensing [13,14]: elastic net logistic regression (ENET), K-nearest neighbor (KNN), support vector machine (SVM), and extreme gradient boosting (XGBoost).

### Self-Supervised Learning Schemes

SSL schemes rely on devising a pretext task, for which a (relatively) large amount of unlabeled data is available, conducive to learning, during a pretraining phase, representations useful to solve the downstream target task [44]. What defines an SSL paradigm is thus its pretext task, consisting of a process, P, to generate pseudo labels and an objective to guide the pretraining. An SSL model typically consists of (1) an encoder EN(x;θ): X->V, learning a mapping from input views 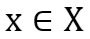 to a representation vector 
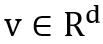
, and (2) a transform head H_ssl_(υ;ξ): V-> Z, projecting the feature embedding into a label space 
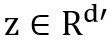
 compatible with the pretext task at hand. When solving the target task, the pretrained encoder EN is retained as a partial solution to the target problem, whereas the pretrained transform head H_ssl_ is discarded and replaced with a new one, H_sl_. Next, EN’s parameter θ may be kept fixed and only H_sl_’s parameters may be learned on the target task. This approach, often referred to as *linear readout* (LR), amounts to treating EN as a frozen feature extractor. Alternatively, instead of just training a new head, the entire network may be retrained on the target task, initializing EN’s parameter θ to the values learned during self-supervised pretraining, a paradigm known as *fine-tuning* (FT). Our SSL models used the same architecture as E4mer, that is, an encoder EN, consisting of convolutional CEs, followed by a transformer RM, and an MLP for the transform head H_ssl_. The success of SSL methods largely comes from designing appropriate pretext tasks that produce representations useful for the downstream target task. This usually involves domain knowledge of the target task. We investigated how different pretext tasks affected downstream performance, experimenting with 2 popular SSL routines that have shown success in other applications: MP and TP.

#### Masked Prediction

This family of SSL methods is characterized by training the model to impute data that have been removed or corrupted by P. It relies on the assumption that context can be used to infer some types of missing information in the data if the domain is well modeled. This strategy was popularized by the huge success of bidirectional encoder representations from transformers (BERT) [38] in NLP applications, and 1 of the first adaptations to multivariate time-series classification was proposed by Zerveas et al [41]. Similar to their implementation, for each segment channel, we sampled a Boolean mask where the sequences of 0s and 1s were sampled from geometric distributions with means of l_0_ and l_1_, respectively, with:



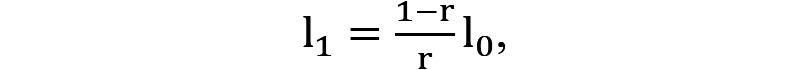



where r is the masking ratio. As shown by Zerveas et al [41], the average length of the 0 sequences (l_m_) and the proportion of masked values (r) were set to 3 seconds and 0.15, respectively. Each segment channel was then multiplied by its corresponding mask, effectively setting to 0 some of the channel-recorded measurements, and inputted to a model that was tasked to recover the original channel values. This was done by minimizing the root mean square error (RMSE) between the masked original value x(t, c) and its reconstruction outputted by the network 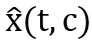:



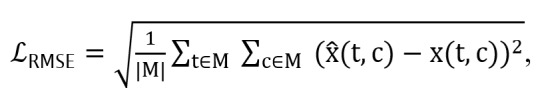



where c and t, respectively, index the channels, and the timestamps of the 0 values in the masks M and |M| are the total number of 0s sampled (ie, the masks’ cardinality).

#### Transformation Prediction

We followed the implementation shown by Wu et al [42], which used SSL for a target task of emotion recognition with E4 recordings. In brief, for each channel, 1 of 6 transformations (ie, identity, Gaussian noise addition, magnitude warping, permutation, time warping, and cropping) was sampled uniformly at random and then applied. The transformed segment was then inputted into a model, which was tasked to guess, for each channel, which of the 6 transformations was applied. This amounted to a multitask, multiclass classification, where the model was trained to minimize channel average categorical cross-entropy (CCE):



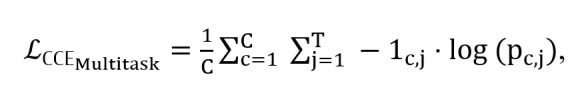



where c indexes the channels and j the transformations, 1_i,j_ is an indicator taking value 1 when j is the correct transformation for channel c and 0 otherwise, and p_c,j_ denotes the predicted probability that transformation j was applied to channel c. By solving this task, Wu et al [42] argued that the model learns representations robust to disturbances in the magnitude and time domains.

### Tuning

A hyperparameter search for all models was carried out with hyperband Bayesian optimization [61]. For the target task, we selected the setting yielding the highest ACC_segment_ in the validation set, whereas in self-supervised pretraining, we selected hyperparameters associated with the lowest relevant loss in the validation pretraining set. Multimedia Appendix 1 shows the hyperparameter search space and the best configuration across all models. Deep learning models were trained with the AdamW optimizer for a maximum of 300 epochs, with a batch size of 256. Moreover, to speed up the training and search procedure, we used an early stopping learning rate scheduler: we reduced the learning rate α_LR_ by a factor of 0.3 if the model did not improve in its validation performance after 10 consecutive epochs, and we terminated the training procedure if the model did not improve after 2 learning rate reductions. Dropout [62] and weight decay were added to prevent overfitting.

### Post hoc Analyses

Toward elucidating key contributors to the viability of SSL, in addition to comparing different pretext task designs, we studied how (1) progressively downsampling unlabeled data sets or (2) removing each data set in turn from the unlabeled collection might impact the performance of our best SSL model. Thus, using the most performative self-supervised scheme, we retrained the SSL model from scratch under configurations (1) and (2) and then tested it on the target task. Note that in both settings, the entire target task training set was kept for pretraining; this is because pretraining on the training set can be always performed at no extra cost in terms of data acquisition. Lastly, we conducted statistical tests to better appreciate how the self-supervised E4mer compared against its fully supervised counterpart and the best-performing CML algorithm and how it was affected by different ablations. Based on whether we considered either (1) recording segments or (2) subjects as our basic analysis units, we had 2 different hypotheses. In (1), we used a linear mixed effects (LME) model to analyze the difference in correct class probabilities between the SSL model and each comparator, considering subjects as a random effect. This accounted for the nested structure of the data, where segments were sampled from individual subjects. A fixed effects intercept was included to test a 0 mean difference between the classifiers at the population level. Additionally, as the ML models we implemented, like most state-of-the-art algorithms [63], effectively treat segments as independent and identically distributed, we used a 2-tailed paired *t* test to assess whether a 0 mean difference in the probability assigned to the correct class was 0. In (2), we checked with a 2-tailed paired *t* test whether the between-classifiers mean difference in the ACC_segment_ by subject was different from 0. To account for multiple testing, within both (1) and (2), a Bonferroni correction was applied. The number of tests was 19, that is, 17 different ablation settings plus 2 tests comparing the best baselines (fully supervised E4mer and the best CML) to SSL.

### Code Used

Python 3.10 programming language was used where deep learning and CML models were implemented in PyTorch [64] and Scikit-learn [65]/XGBoost [66] respectively, while hyperparameter tuning was performed in both cases with weights and biases [67]. The best hyperparameter setting found during tuning for each model is reported in Multimedia Appendix 1. All deep learning models were trained on a single Nvidia A100 graphical processing unit (GPU).

### Ethical Considerations

The TIMEBASE/INTREPIBD study was conducted in accordance with the ethical principles of the Declaration of Helsinki and Good Clinical Practice and the Hospital Clinic Ethics and Research Board (HCB/2021/104). All participants provided written informed consent prior to their inclusion in the study. All data were collected anonymously and stored encrypted in servers complying with the General Data Protection Regulation (GDPR) and the Health Insurance Portability and Accountability Act (HIPAA). Regarding other studies included in this work, we referred to relevant publications.

## Results

### Surrogate Tasks Used in Self-Supervised Pretraining

**Figure 3:**
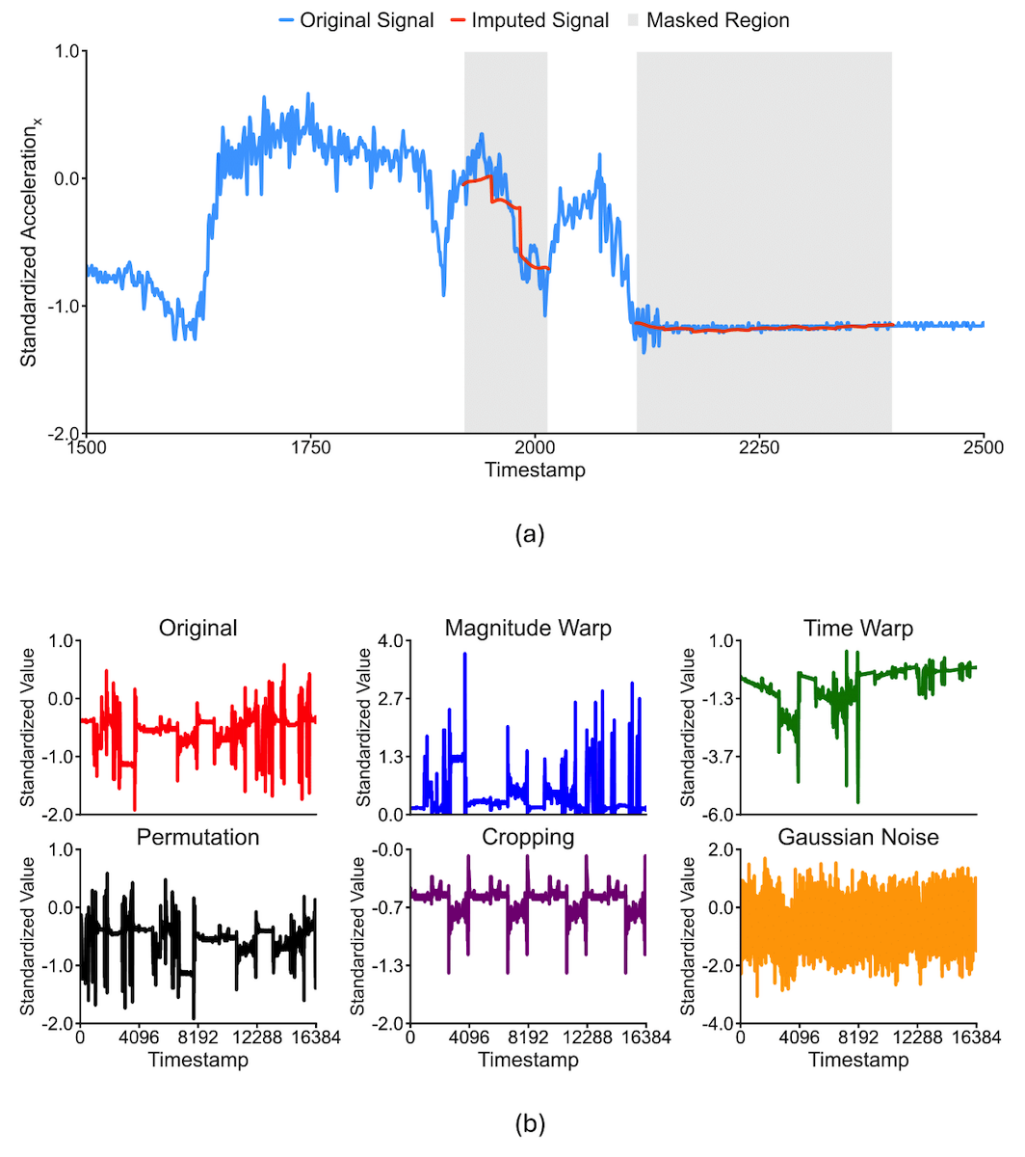


The same model, using the E4mer architecture (Figure 2), was used across different pretext tasks. Figure 3 illustrates the surrogate tasks we experimented with. In MP (Figure 3a), parts of the input segments were zeroed out by multiplication with a Boolean mask sampled, as shown by Zerveas et al [41], and the model was trained to recover the original input segments. Although the model output entire segments, only the masked values were considered toward the loss computation, that is, the RMSE. The assumption was that the model acquires good representations of the underlying structure of the data when learning to solve this task. Our best model had an error of 0.1347 on the test set (notice that input segments were channel-wise standardized).

In TP (Figure 3b), 1 transformation was sampled from a set and applied to each channel independently, and the model learned which transformation each channel underwent, minimizing the channel average CCE. We used the same transformations as Wu et al [42], who experimented with an E4 for a downstream task of emotion recognition. The rationale was to encourage robustness against signal disturbances introduced with the transformations. The test loss of the selected model was 0.5000.

### Target Task Performance Comparison

Table 2 illustrates the performance under each model we developed. Although they were all optimized for segment ACC, we also reported subject ACC since in a clinical scenario, a decision needs to be made at the subject level. Note that although ACC was a suitable metric in our use case as data were perfectly balanced, we also provided complementary metrics (precision, recall, *F*_1_-score, and area under the receiver operating characteristic curve [AUROC]), both at the segment and at the patient level. At the subject level, the predicted class was the result of a majority vote over that subject’s segments, while the predicted probabilities under each class were derived by summing segments’ predicted probabilities for that subject and normalizing by the corresponding segment number. MP self-supervised pretraining comfortably outperformed end-to-end SSL, while also surpassing other self-supervised approaches.

**Table 2:** Performance in differentiating an acute MD^a^ episode from euthymia across different models.

Model		ACC^b^			Precision			Recall			*F*_1_ score			AUROC^c^	
		Segment	Subject	Segment		Subject	Segment		Subject	Segment		Subject	Segment		Subject
**SL^d^**															
	ENET^e^	66.38	71.88	66.22		75	66.86		65.63	66.54		70	72.24		82.25
	KNN^f^	70.37	82.81	69.09		80	73.74		81.2	71.34		80.6	73.27		83.26
	SVM^g^	71.25	81.25	71.87		80	71.40		77.65	71.63		78.81	73.44		83.21
	XGBoost^h^	72.02	82.81	71.33		83	72.11		81.1	71.72		82.03	72.44		83.17
	E4mer^i^	75.35	81.25	73.46		80.55	75.34		82.14	74.39		81.33	75.68		82.22
**SSL^j^**															
	MP^k^ (LR^l^)	77.53	87.5	78.34		88.6	77.41		88	77.87		88.3	78.02		89.2
	MP (FT^m^)	81.23^n^	90.63^n^	80.91^n^		90.11^n^	82.00^n^		92.87^n^	81.45^n^		91.47^n^	82.02^n^		93.11^n^
	TP^o^ (LR)	71.16	81.25	72.12		82.44	72.01		82.31	72.06		82.37	71.89		84.12
	TP (FT)	75.69	84.38	75.41		82.11	74.79		83.9	75.1		83	75.21		84.23

^a^MD: mood disorder.

^b^ACC: accuracy.

^c^AUROC: area under the receiver operating characteristic curve.

^d^SL: supervised learning.

^e^ENET: elastic net logistic regression.

^f^KNN: K-nearest neighbor.

^g^SVM: support vector machine.

^h^XGBoost: extreme gradient boosting.

^i^E4mer: E4-tailored transformer.

^j^SSL: self-supervised learning

^k^MP: masked prediction.

^l^LR: linear readout.

^m^FT: fine-tuning.

^n^The best results.

^o^TP: transformation prediction.

The E4mer and CML baselines performed to a similar level: although E4mer was superior to XGBoost in terms of ACC_segment_ (75.35 vs 72.02), it was trumped by CML on ACC_subject_ (82.81 vs 81.25). Other CML baselines fared worse than XGBoost. MP pretraining led to a target task performance, substantially higher than the baselines, under both metrics. Although both LR and FT dominated over supervised learning (SL), the latter scored the highest performance with ACC_segment_ and ACC_subject_ of 0.8123 and 0.9063, respectively. However, TP led to only modest improvement over E4mer. Statistical tests comparing the best SSL scheme (ie, MP with FT) against the fully supervised E4mer and XGBoost were significant at both the segment and the subject level. In particular, comparison with E4mer yielded *P*_Bonferroni_ values of .03 for the LME model and <.001 and .02 for the *t* test at the segment and the subject level, respectively. For XGBoost, *P*_Bonferroni_ values were .04 for the LME model and <.01 and .01 for the *t* test at the segment and the subject level, respectively.

**Figure 4:**
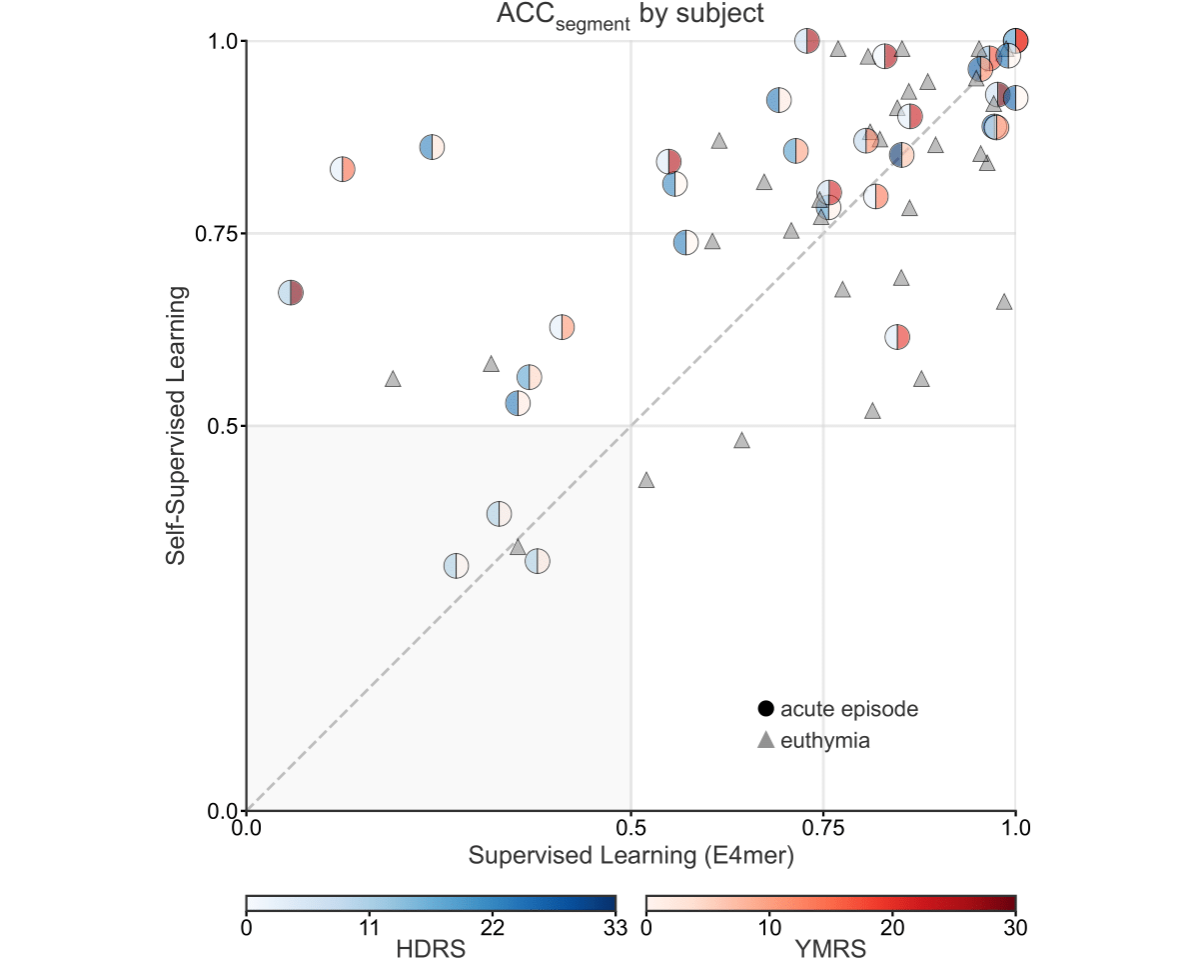
SSL beats SL by 4 (9.4%) more correctly classified subjects. ACCsegment under SSL and SL (E4mer) within each subject’s test segments: subjects in the euthymia group are represented as triangles, while subjects on an acute affective episode are shown as circles with the left half colored in blue and the right half in red, with a gradient proportional to the total sum on the HDRS and the YMRS, respectively. Subjects’ position on the x and y axes corresponds to their proportion of recording segments correctly classified by SL and SSL, respectively. Note that a subject’s majority vote over their segments is in agreement with the subject’s true mood state when the proportion of correctly classified segments from that subject is greater than 0.5. The HDRS and the YMRS range shown on the color bar refer to values scored in the TIMEBASE/INTREPIBD sample, while the total score, in general, range is 0-52 and 0-60, respectively. ACCsegment: segment accuracy; E4mer: E4-tailored transformer; HDRS: Hamilton Depression Rating Scale-17; SL: supervised learning; SSL: self-supervised learning; TIMEBASE/INTREPIBD: Identifying Digital Biomarkers of Illness Activity in Bipolar Disorder/Identifying Digital Biomarkers of Illness Activity and Treatment Response in Bipolar Disorder.

Comparison of the best SSL with its SL counterpart in terms of ACC_segment_ by subject (Figure 4) suggested that only 2 (3.1%) patients with euthymia were misclassified by SSL but correctly classified by the supervised E4mer. However, SL mispredicted 8 (12.5%) individuals that SSL got right. Patients on an acute MD episode are shown as dots with a color gradient proportional to their total score on the HDRS [46] (left half) and the YMRS [47] (right half), 2 clinician-administered questionnaires tracking depression and mania severity, respectively. Subjects on an acute MD episode misclassified by SL included patients with severe depressive (or manic) symptomatology. Notably, both SSL and SL failed in the case of 4 (6.3%) subjects, including 3 (75%) patients on an acute MD episode with relatively moderate severity.

### Ablation Analyses and Learned Representations

Tables 3 and 4 show the difference in the target task ACC_segment_ and ACC_subject_ resulting from pretraining the best SSL on parts of the unlabeled data collection and then FT it onto the target task. Ablation analyses showed a positive trend between unlabeled data availability and target task performance, but data set–specific unobserved factors likely played a role. The difference in ACC_segment_ and ACC_subject_ from pretraining on just parts of the entire unlabeled data collection is shown in the tables. An LME model and a 2-tailed paired *t* test assessed whether the mean difference in predicted probabilities for the segment’s correct class differed from 0, with the former correcting for subjects as a random effect. A 2-tailed paired *t* test assessed whether the mean difference in the number of correctly classified segments by subject differed from 0. In each test, the comparator was the best-performing self-supervised model. P values are corrected with Bonferroni’s method. Note that a majority vote over a subject’s segments was used to issue subject-level predictions, and ACC_subject_ was simply the fraction of correct majority votes in the test set. ACC_subject_, therefore, did not consider the proportion of votes over a subject’s segments in favor of the subject’s correct class but just whether a majority, no matter how small or large, was reached in agreement with the correct class. However, the *t* test (subject) assessed a 0 mean difference in the proportion of votes, within subjects, for the correct class. As shown in Table 3, self-supervised pretraining, preceding FT on the target task, therefore used only a fraction of the total unlabeled collection. A resampling ratio of 0% meant that self-supervised pretraining was performed on the target training set only.

The Pearson correlation coefficient (PCC) between unlabeled data downsampling ratios and the difference in ACC_segment_ and ACC_subject_ was 0.9401 and 0.9449, respectively, indicating a strong dependence between performance and unlabeled data availability. Similarly, excluding individual data sets from pretraining impacted ACC_segment_ and ACC_subject_ proportionally to their relative size (PCC=–0.8185 and –0.4083, respectively). Notably, however, TIMEBASE/INTREPIBD, despite being collected at the same site as the target task data and making up the largest share of the unlabeled data collection, did not leave the largest dent in performance when excluded from training. Furthermore, excluding some data sets resulted in performance improvement. Differences in ACC_segment_ and ACC_subject_ did not always have the same sign because of the way they were defined. Indeed, it is, for example, possible that the absolute number of correctly classified segments decreased but enough previously misclassified segments within a subject were now correctly classified so that the majority vote for that subject flipped. Statistical analyses showed that the ablation of a single data set was associated with nonsignificantly different performance in terms of correctly classified segments within subjects. At the level of the probability assigned to the correct class for each segment, LME results were significant only for a data set, whereas results were mixed for *t* tests. Stratified resampling gave positive results, but the significance for LME was reached only at lower downsampling ratios.

**Figure 5:**
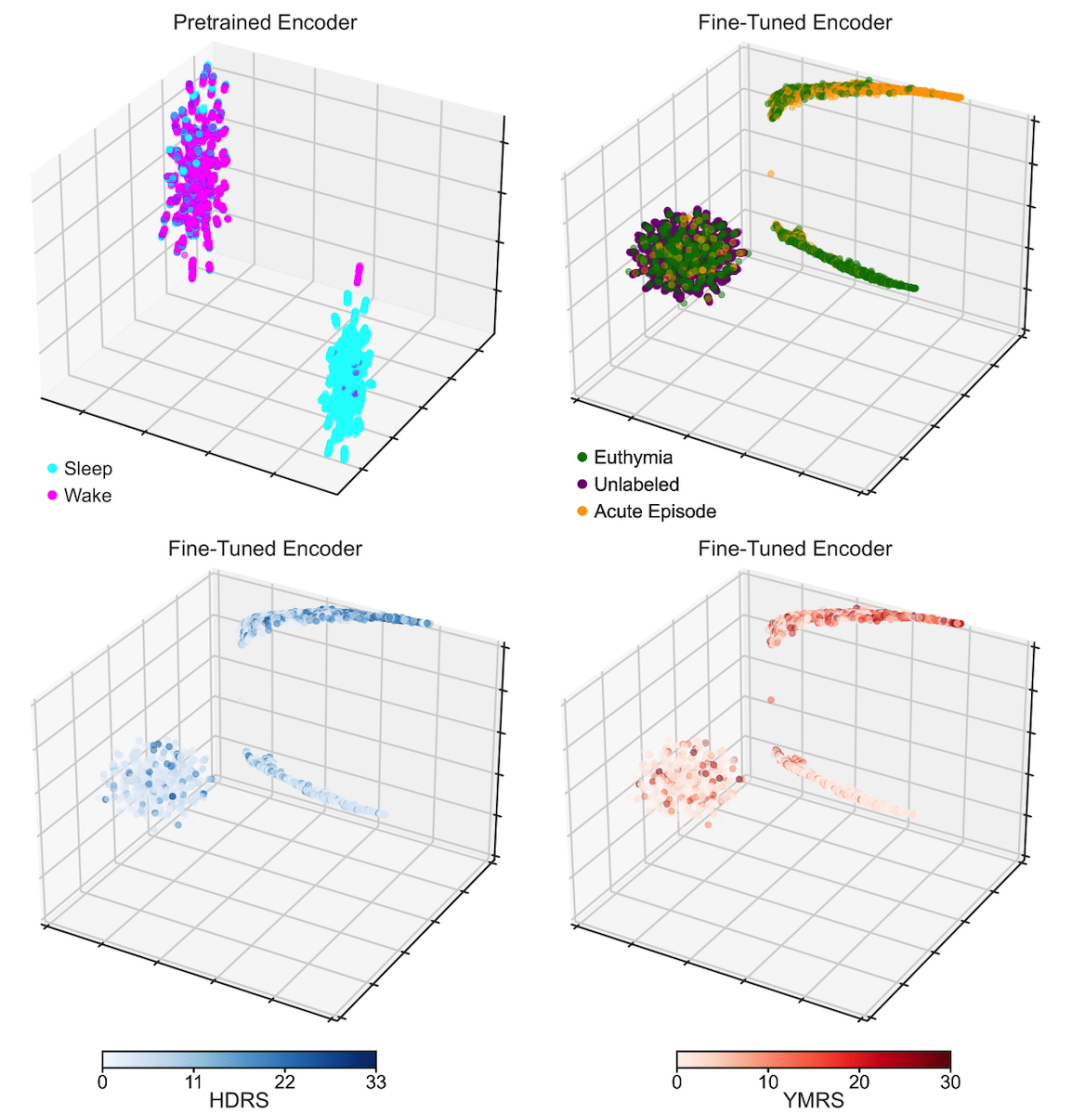
Reassuringly, the learned embeddings seem to have captured meaningful semantics about the underlying context. (Top left) Embeddings from the encoder pretrained on MP map sleep and wake segments to different parts of the latent space. (Top right) Embeddings from the encoder FT on the target task show that segments from the unlabeled open access data sets, which presumably do not contain subjects on an acute MD episode, tend to cluster with part of the segments from patients in euthymia. Embeddings from the fine-tuned encoder show a gradient in symptoms’ severity across target task segments, as revealed by (bottom left) the HDRS and (bottom right) the YMRS total score. Note that unlabeled segments are not shown in the bottom left or right plot and that the HDRS and YMRS ranges shown on the color bar refer to values scored in the TIMEBASE/INTREPIBD sample, while the total score range, in general, can be 0-52 and 0-60, respectively. FT: fine-tuning; HDRS: Hamilton Depression Rating Scale-17; MD: mood disorder; MP: masked prediction; TIMEBASE/INTREPIBD: Identifying Digital Biomarkers of Illness Activity in Bipolar Disorder/Identifying Digital Biomarkers of Illness Activity and Treatment Response in Bipolar Disorder; YMRS: Young Mania Rating Scale.

Lastly, we visualized the representations learned by the encoder, EN, part of our best-performing models to gain further insights. As EN’s output had dimensionality (B=number of segments, N=number of timestamps, D=transformer’s model dimension), for visualization purposes, we averaged out the D axis and then used Uniform Manifold Approximation and Projection (UMAP) [68], a powerful nonlinear dimensionality reduction technique, to embed the resulting N-dimensional data points into 3 dimensions. The top-left plot of Figure 5 shows the representations learned during self-supervised pretraining with MP. The segments shown are the target task test segments, along with an equal number of segments belonging to the same sessions but taken from the sleep state, which the SSL model was never exposed to during training. Wake and sleep segments have different embeddings, suggesting that the model captured this structure in the physiological data: a Gaussian mixture model, indeed, recovered 2 clusters, one with predominantly sleep segments (n=4081, 82.66%) and the other with the majority of wake segments (n=3272, 95.58%). It should be noted that sleep and wake naturally have quite different semantics with respect to physiological data, and the algorithm we used for sleep/wake differentiation (Van Hees [52]) uses a simple heuristic defining sleep as a sustained lack of significant changes in the acceleration angle. The top-right and bottom plots of Figure 5 illustrate the representations from the SSL model upon FT on the target task. The top-right scatter plot displays the target task test segments, as well as pretraining validation set segments (except for the pretraining segments from the TIMEBASE/INTREPIBD collection). The latter group of segments we assumed as being taken from subjects without an acute MD episode and, arguably, most even without any historical MD diagnosis, since the open access data sets we found did not select for patients with an MD. The plot shows 3 clusters whose composition, as recovered with a Gaussian mixture model, was as follows: (1) n=1464 (79.26%) acute MD episode and n=383 (20.7%) euthymia; (2) n=1120 (74.16%) euthymia and n=390 (25.84%) acute MD episode; and (3) n=7801 (91.01%) unlabeled segments, n=683 (7.96%) euthymia, and n=88 (1.02%) acute MD episode. The bottom plots in Figure 5 show target task segments test segments only (no unlabeled segment), colored with a gradient proportional to symptoms’ severity, as assessed with the HDRS [46] and the YMRS [47]. Embeddings would seem to suggest a progression in symptoms’ severity across the 2 clusters of segments on the right of the scatter plot.

**Table 3:** Ablation analyses results: the unlabeled collection was downsampled, stratifying by data sets.

Resampling ratio	80%	60%	40%	20%	0%
ACC^a^_segment_ difference	–0.23^b^	–2.14^b^	–6.07^b^	–6.35^b^	–7.07^b^
ACC_subject_ difference	–1.57^b^	–1.57^b^	–4.70^b^	–4.70^b^	–7.82^b^
LME^c^ *P* value	.09	.07	.06	.05	.04
*t* Test (segment) *P* value	<.001	<.001	<.001	<.001	<.001
*t* Test (subject) *P* value	.001	.001	.001	.001	.001

^a^ACC: accuracy.

^b^Deterioration in performance upon retraining on the ablated unlabeled data collection.

^c^LME: linear mixed effects.

**Table 4:** Ablation analyses results: self-supervised pretraining was conducted, leaving out each data set in turn from the unlabeled collection.

Data set	Relative size	ACC^a^_segment_ difference	ACC_subject_ difference	LME^b^ *P* value	*t* Test (segment) *P* value	*t* Test (subject) *P* value
Alcohol and Drug Abuse Research Program (ADARP)	12.34	–2.44^c^	–1.57^c^	.01	<.001	.99
Stress Predict	0.30	–0.21^c^	–1.57^c^	.23	.99	.99
Toadstool	0.04	0.52^d^	–3.13^c^	.99	.99	.99
Unlabeled Empatica E4 Wristband (UE4W)	2.32	–1.93^c^	–4.70^c^	.99	.003	.99
Wearable Human Energy Expenditure Estimation (WEEE)	0.18	1.19^d^	1.57^d^	.99	.90	.99
Wearable Stress and Affect Detection (WESAD)	0.42	–0.51^c^	–1.57^c^	.99	.99	.99
Wearable Exam Stress Dataset (WESD)	0.72	1.90^d^	1.57^d^	.06	.05	.99
In-GaugeEn-Gage	17.55	–4.44^c^	–4.70^c^	.99	<.001	.63
Nurse Stress Detection	11.82	–0.81^c^	–1.57^c^	.99	.99	.99
BIG IDEAs Lab	19.38	–2.09^c^	–1.57^c^	.99	.08	.99
PPG Dataset for Motion Compensation and Heart Rate Estimation in Daily Life Activities (PPG-DaLiA)	0.69	1.93^d^	4.70^d^	.53	<.001	.99
TIMEBASE/INTREPIBD^e^	34.24	–4.32^c^	–3.13^c^	.99	.99	.38

^a^ACC: accuracy.

^b^LME: linear mixed effects.

^c^Deterioration in performance upon retraining on the ablated unlabeled data collection.

^d^Improvement in performance upon retraining on the ablated unlabeled data collection.

^e^TIMEBASE/INTREPIBD: Identifying Digital Biomarkers of Illness Activity in Bipolar Disorder/Identifying Digital Biomarkers of Illness Activity and Treatment Response in Bipolar Disorder.

## Discussion

### Principal Findings

Personal sensing is likely to play a key role in health care supply, creating unprecedented opportunities for patient monitoring and just-in-time adaptive interventions [69]. Toward delivering on this promise, expert annotation is a major obstacle; this is especially the case with MDs, wherein data annotation is particularly challenging and time-consuming, considering the nature of the disorders.

To the best of our knowledge, we are the first to show that SSL is a viable paradigm in personal sensing for MDs, mitigating the annotation bottleneck, thanks to the repurposing of existing unlabeled data collected in settings as different as subjects playing Super Mario [27], taking university exams [29], or performing physical exercise [28].

We took on a straightforward yet fundamental task, that is, to distinguish acute MD episodes from euthymia. Timely recognition of an impending MD episode in someone with a historical MD diagnosis regardless of the episode polarity (depressive, manic, or mixed) may, indeed, enable preemptive interventions and better outcomes [5]. Our results suggest that with a sample size on the order of magnitude that is typical of studies into personal sensing for MDs, a modern deep learning fully supervised pipeline (E4mer) may offer no substantial improvements over simpler CML algorithms (eg, XGBoost), despite higher development and computational costs. However, the accumulation and repurposing of existing unlabeled data sets for an SSL pretraining phase leads to a confident margin of improvement: ACC_segment_ and ACC_subject_ improve by 7.8% and 11.54%, respectively, relative to the fully supervised E4mer, with 6 (9.4%) of 64 more subjects correctly classified.

Our findings further show that careful choice of the pretext task, as well documented in the literature on SSL [40], is key toward learning useful representations for the downstream target task. Unlike MP, improvement, if any at all, from TP was only modest. This is not to say that such a pretext task may in general fail to deliver on acute MD episode versus euthymia differentiation. Indeed, the specific transformations we implemented, borrowed from Wu et al [42], may have been suboptimal for our downstream task, pointing to the importance of domain knowledge (including clinical expertise) in pretext task design. Lastly, although SSL relaxes dependence on large, annotated data sets, our results indicate that its success relies on the size of unlabeled data. Ablation analyses, indeed, showed a positive correlation between target task performance and the size of the corpus available for pretraining. Data set–idiosyncratic factors accounting for the nonperfect correlation between the relative size and impact on target task performance may be present. Speculatively, these may include noise in the data, (dis)similarity of recording conditions, or (ir)relevance for the target task of the representations learned modeling the domain of the unlabeled data set.

Statistical analyses showed that excluding from pretraining any of the individual unlabeled data sets, while keeping all others, is not associated with a significant change in performance on the proportion of correctly classified segments within subjects. The lack of a significant effect in either direction (improvement or deterioration), along with a significantly superior performance of SSL over fully supervised schemes, indicate that pretraining on big data collections leads to higher performance than taking on the target task from scratch. Of importance, adding data sets for pretraining from domains not immediately related to the target task did not undermine the model. Pretraining under progressively lower downsampling ratios lent further support to the importance of data size. This is consistent with the deep learning recipe where the bigger the pretraining corpus, the better the results [70]. Results from tests at the level of segment-predicted probabilities are consistent with this view. Of the data sets comprising less than 1% of the entire unlabeled collection, only 1 reached statistical significance. LME has more flexibility to explain the data since rather than pooling all segments together in a unique (bigger) population, it treats them as embedded within subjects. This explains the lack of statistical significance relative to the *t* tests under various data ablation regimes.

### Limitations

We acknowledge the following limitations of this study. We deliberately chose the simplest task that has clinical relevance in personal sensing for MDs since our focus was on SSL; however, we appreciate that a more fine-grained MD description, beyond a simple acute MD episode versus euthymia binary classification, may add further clinical value [35]. As the literature on SSL is expanding at a fast pace, a thorough search of different approaches was beyond the scope of this work. We acknowledge that other pretext tasks can be deployed, and although the architectural choice may have an impact on SSL, we settled for just 1 reasonable, modern model design with a transformer [43] as a workhorse for representation learning. Lastly, given the naturalist design of the study, reflective of the intended use of personal sensing in a clinical setting, we could not exclude the effect of confounders, including medications, on the physiological variables. However, we reported medication classes administered in the cohort and verified a lack of any significant association between target classes (euthymia vs acute MD episode) and being on a given medication class.

### Future Directions

As our findings indicate that the choice of the pretext task has a significant impact on target task performance, further efforts should be put into pretext task design. Indeed, although MP is a general-purpose strategy inspired by the great success of BERT [38] in NLP, the literature on SSL [40] suggests that domain knowledge may help tailor the pretext task to the specific use case. A promising approach we did not explore is contrastive learning [71], which, indeed, relies on domain knowledge of how augmented views of the input are created, especially since most experience today is in computer vision and NLP, while physiological multivariate time series are relatively unexplored.

### Conclusion

This work shows that SSL is a promising paradigm for mitigating the annotation bottleneck, 1 of the major barriers to the development of artificial intelligence–powered clinical decision support systems using personal sensing to help monitor MDs, thus enabling early intervention. The collection and preprocessing of open access unlabeled data sets that we curated (E4SelfLearning) can foster future research into SSL, therefore advancing the translation of personal sensing into clinical practice.

## Appendix

Multimedia Appendix 1 Supplementary material.

## Abbreviations

ACC: accuracy
AUROC: area under the receiver operating characteristic curve
BD: bipolar disorder
BVP: blood volume pressure
CCE: categorical cross-entropy
CE: channel embedding
CML: classical machine learning
DSM-5: Diagnostic and Statistical Manual, Fifth Edition
E4mer: E4-tailored transformer
EDA: electrodermal activity
ENET: elastic net logistic regression
FT: fine-tuning
HDRS: Hamilton Depression Rating Scale-17
HR: heart rate
IBI: interbeat interval
KNN: K-nearest neighbor
LME: linear mixed effects
LR: linear readout
MD: mood disorder
MDD: major depressive disorder
ML: machine learning
MLP: multilayer perceptron
MP: masked prediction
NLP: natural language processing
PCC: Pearson correlation coefficient
RM: representation module
RMSE: root mean square error
SL: supervised learning
SSL: self-supervised learning
SVM: support vector machine
TEMP: skin temperature
TIMEBASE/INTREPIBD: Identifying Digital Biomarkers of Illness Activity in Bipolar Disorder/Identifying Digital Biomarkers of Illness Activity and Treatment Response in Bipolar Disorder
TP: transformation prediction
XGBoost: extreme gradient boosting
YMRS: Young Mania Rating Scale

## Data Availability

The E4SelfLearning collection is available at Reference [72], and the codebase is available at Reference [73]. Data in deidentified form from the Identifying Digital Biomarkers of Illness Activity in Bipolar Disorder/Identifying Digital Biomarkers of Illness Activity and Treatment Response in Bipolar Disorder study may be made available from the corresponding author upon reasonable request.
