# Supplementary material for: Wearable Data From Subjects Playing Super Mario, Taking University Exams, or Performing Physical Exercise Help Detect Acute Mood Disorder Episodes via Self-Supervised Learning: Prospective, Exploratory, Observational Study
Source: JMIR Mhealth Uhealth. 2024 Jul 17;12:e55094. doi: 10.2196/55094 (PMC11292167; doi:10.2196/55094)
Supplement: Multimedia Appendix 1 [file mhealth_v12i1e55094_app1.doc]

**Supplementary Material**

Table S1 **Details on the datasets used in the present study.** N: subjects #; H_w_: waking hours # (rounded); H_s_: sleeping hours # (rounded); H_o_: off-body hours # (rounded); ^†^ marks datasets that constitute the E4SelfLearning collection which we make publicly available.

| **Dataset** | **N** | **H_w_ (segments #)** | **H_s_** | **H_o_** |
| --- | --- | --- | --- | --- |
| **ADARP^†^** | 11 | 773 (14666) | 109 | 525 |
| **Stress Predict^†^** | 26 | 18 (358) | 1 | 3 |
| **Toadstool^†^** | 9 | 3 (52) | 3 | 0 |
| **UE4W^†^** | 1 | 128 (2753) | 109 | 14 |
| **WEEE^†^** | 17 | 12 (216) | 2 | 1 |
| **WESAD^†^** | 15 | 23 (504) | 3 | 2 |
| **WESD^†^** | 9 | 35 (856) | 0 | 52 |
| **In-GaugeEn-Gage^†^** | 27 | 1010 (20856) | 14 | 696 |
| **Nurse Stress Detection^†^** | 15 | 685 (14040) | 24 | 364 |
| **BIG IDEAs Lab^†^** | 16 | 1218 (23025) | 805 | 583 |
| **PPG-DaLiA^†^** | 15 | 33 (815) | 1 | 2 |
| **TIMEBASE/INTREPIBD unlabelled** | 91 | 2316 (40686) | 2320 | 525 |
| **TIMEBASE/INTREPIBD target task** | 64 | 1512 (Train: 18896, Val: 3904, Test:4128) | 1182 | 523 |

Table S2 **Treatment status across the acute episode and euthymia classes.** The percentage of patients across the two classes, i.e. acute episode and euthymia, receiving a given treatment is shown. Chi-squared tests (with Bonferroni correction) were carried out to check associations between disease status (acute episode or euthymia) and treatment status (drug being administered or not administered). No test yielded a significant p-value. Li: lithium; SSRI: selective serotonin reuptake inhibitors; SNRI: serotonin and norepinephrine reuptake inhibitors; TCA: tricyclics; MAOI: monoamine oxidase inhibitors; OAD: other antidepressants; AP1: first generation antipsychotic; AP2 second-generation antipsychotic; AED: antiepileptic drug; AMP: amphetamines; AH: antihistamines; AAD: antiarrhythmic drug; AC: other anticholinergic medications; BDZ: benzodiazepines.

|  | **Li** | **SSRI** | **SNRI** | **TCA** | **MAOI** | **OAD** | **AP1** | **AP2** |
| --- | --- | --- | --- | --- | --- | --- | --- | --- |
| **Euthymia** | 46.88 | 34.37 | 12.5 | 12.5 | 0 | 15.62 | 6.25 | 50.00 |
| **Acute Episode** | 55.55 | 11.11 | 25.92 | 18.51 | 0 | 22.22 | 3.70 | 70.37 |

|  | **AED** | **β-blocker** | **Opioid** | **AMP** | **AH** | **AAD** | **AC** | **BDZ** |
| --- | --- | --- | --- | --- | --- | --- | --- | --- |
| **Euthymia** | 50.00 | 9.37 | 0 | 0 | 0 | 0 | 3.12 | 39.6 |
| **Acute Episode** | 29.63 | 3.70 | 0 | 0 | 0 | 0 | 3.7 | 50.07 |

**Hyperparameters Tuning**

Table S3 **Masked Prediction**

| **HYPERPARAMETER** | **SEARCH SPACE** | **FINAL VALUE** |
| --- | --- | --- |
| LEARNING RATE α_LR_ | UNIFORM, MIN: 0.0001, MAX: 0.01 | 0.009 |
| WEIGHT DECAY | UNIFORM, MIN: 0, MAX: 1 | 0.0572 |
| **CHANNEL EMBEDDINGS** |  |  |
| NUM. FILTERS | CATEGORICAL, 2^i^ FOR i ∈ {2, 3, 4} | 2^4^ |
| **REPRESENTATION** **MODULE** |  |  |
| D MODEL | CATEGORICAL, 2^i^ FOR i ∈ {5, 6, 7, 8, 9} | 2^8^ |
| NUM. HEADS | UNIFORM, MIN: 1, MAX: 4 | 2 |
| NUM. BLOCKS | UNIFORM, MIN: 1, MAX: 4 | 4 |
| ATTENTION DROPOUT | UNIFORM, MIN: 0, MAX: 0.5 | 0.2699 |
| DROP PATH | UNIFORM, MIN: 0, MAX: 0.5 | 0.0034 |
| MLP DIM. | UNIFORM, MIN: 32, MAX: 256, INTERVAL: 8 | 72 |
| MLP DROPOUT | UNIFORM, MIN: 0, MAX: 0.5 | 0.0824 |
| DISABLE BIAS | BERNOULLI | 1 |

Table S4 **Transformation Prediction**

| **HYPERPARAMETER** | **SEARCH SPACE** | **FINAL VALUE** |
| --- | --- | --- |
| LEARNING RATE α_LR_ | UNIFORM, MIN: 0.0001, MAX: 0.01 | 0.009 |
| WEIGHT DECAY | UNIFORM, MIN: 0, MAX: 1 | 0.101 |
| **CHANNEL EMBEDDINGS** |  |  |
| NUM. FILTERS | CATEGORICAL, 2^i^ FOR i ∈ {2, 3, 4} | 2^4^ |
| **REPRESENTATION** **MODULE** |  |  |
| D MODEL | CATEGORICAL, 2^i^ FOR i ∈ {5, 6, 7, 8, 9} | 2^9^ |
| NUM. HEADS | UNIFORM, MIN: 1, MAX: 4 | 2 |
| NUM. BLOCKS | UNIFORM, MIN: 1, MAX: 4 | 1 |
| ATTENTION DROPOUT | UNIFORM, MIN: 0, MAX: 0.5 | 0.3883 |
| DROP PATH | UNIFORM, MIN: 0, MAX: 0.5 | 0.1783 |
| MLP DIM. | UNIFORM, MIN: 32, MAX: 256, INTERVAL: 8 | 176 |
| MLP DROPOUT | UNIFORM, MIN: 0, MAX: 0.5 | 0.0310 |
| DISABLE BIAS | BERNOULLI | 1 |

Table S5 **Masked Prediction, Linear Readout**

| **HYPERPARAMETER** | **SEARCH SPACE** | **FINAL VALUE** |
| --- | --- | --- |
| LEARNING RATE α_LR_ | UNIFORM, MIN: 0.0001, MAX: 0.01 | 0.0058 |
| WEIGHT DECAY | UNIFORM, MIN: 0, MAX: 1 | 0.8189 |

Table S6 **Transformation Prediction, Linear Readout**

| **HYPERPARAMETER** | **SEARCH SPACE** | **FINAL VALUE** |
| --- | --- | --- |
| LEARNING RATE α_LR_ | UNIFORM, MIN: 0.0001, MAX: 0.01 | 0.0058 |
| WEIGHT DECAY | UNIFORM, MIN: 0, MAX: 1 | 0.7952 |

Table S7 **Masked Prediction, Fine-tuning**

| **HYPERPARAMETER** | **SEARCH SPACE** | **FINAL VALUE** |
| --- | --- | --- |
| LEARNING RATE α_LR_ | UNIFORM, MIN: 0.0001, MAX: 0.01 | 0.0010 |
| WEIGHT DECAY | UNIFORM, MIN: 0, MAX: 1 | 0.0232 |
| **REPRESENTATION** **MODULE** |  |  |
| ATTENTION DROPOUT | UNIFORM, MIN: 0, MAX: 0.5 | 0.4732 |
| DROP PATH | UNIFORM, MIN: 0, MAX: 0.5 | 0.0103 |
| MLP DROPOUT | UNIFORM, MIN: 0, MAX: 0.5 | 0.1209 |

Table S8 **Transformation Prediction, Fine-tuning**

| **HYPERPARAMETER** | **SEARCH SPACE** | **FINAL VALUE** |
| --- | --- | --- |
| LEARNING RATE α_LR_ | UNIFORM, MIN: 0.0001, MAX: 0.01 | 0.0011 |
| WEIGHT DECAY | UNIFORM, MIN: 0, MAX: 1 | 0.7052 |
| **REPRESENTATION** **MODULE** |  |  |
| ATTENTION DROPOUT | UNIFORM, MIN: 0, MAX: 0.5 | 0.0754 |
| DROP PATH | UNIFORM, MIN: 0, MAX: 0.5 | 0.2065 |
| MLP DROPOUT | UNIFORM, MIN: 0, MAX: 0.5 | 0.2782 |

Table S9 **E4mer fully-supervised**

| **HYPERPARAMETER** | **SEARCH SPACE** | **FINAL VALUE** |
| --- | --- | --- |
| LEARNING RATE α_LR_ | UNIFORM, MIN: 0.0001, MAX: 0.01 | 0.0052 |
| WEIGHT DECAY | UNIFORM, MIN: 0, MAX: 1 | 0.0016 |
| **CHANNEL EMBEDDINGS** |  |  |
| NUM. FILTERS | CATEGORICAL, 2^i^ FOR i ∈ {2, 3, 4} | 2^2^ |
| **REPRESENTATION** **MODULE** |  |  |
| D MODEL | CATEGORICAL, 2^i^ FOR i ∈ {5, 6, 7, 8, 9} | 2^5^ |
| NUM. HEADS | UNIFORM, MIN: 1, MAX: 4 | 2 |
| NUM. BLOCKS | UNIFORM, MIN: 1, MAX: 4 | 4 |
| ATTENTION DROPOUT | UNIFORM, MIN: 0, MAX: 0.5 | 0.1702 |
| DROP PATH | UNIFORM, MIN: 0, MAX: 0.5 | 0.4676 |
| MLP DIM. | UNIFORM, MIN: 32, MAX: 256, INTERVAL: 8 | 120 |
| MLP DROPOUT | UNIFORM, MIN: 0, MAX: 0.5 | 0.1037 |
| DISABLE BIAS | BERNOULLI | 0 |

Table S10 **XGBoost**

| **HYPERPARAMETER** | **SEARCH SPACE** | **FINAL VALUE** |
| --- | --- | --- |
| COLSAMPLE BY TREE | UNIFORM, MIN: 0.1, MAX: 1 | 0.9639 |
| GAMMA | UNIFORM, MIN: 0, MAX: 10 | 0.7854 |
| LEARNING RATE | UNIFORM, MIN: 0.001, MAX: 0.3 | 0.2848 |
| MAX DEPTH | UNIFORM, MIN: 3, MAX: 10, INTERVAL: 1 | 9 |
| MIN CHILD WEIGHT | UNIFORM, MIN: 0.01, MAX: 10 | 8.7346 |
| N ESTIMATORS | UNIFORM, MIN: 5, MAX: 50, INTERVAL: 1 | 50 |
| REG ALPHA | UNIFORM, MIN: 0, MAX: 10 | 0.5811 |
| REG LAMBDA | UNIFORM, MIN: 0, MAX: 10 | 4.2859 |
| SUBSAMPLE | UNIFORM, MIN: 0, MAX: 1 | 0.9424 |

Table S11 **SVM**

| **HYPERPARAMETER** | **SEARCH SPACE** | **FINAL VALUE** |
| --- | --- | --- |
| C | UNIFORM, MIN: 0.1, MAX: 100 | 9.639 |
| GAMMA | UNIFORM, MIN: 0.1, MAX: 10 | 40.62 |
| DEGREE | DISCRETE UNIFORM, MIN: 2, MAX: 10 | 3 |
| KERNEL | CATEGORICAL: “linear”, ”poly”, ”rbf”, ”sigmoid” | ”poly” |

Table S12 **KNN**

| **HYPERPARAMETER** | **SEARCH SPACE** | **FINAL VALUE** |
| --- | --- | --- |
| N_NEIGHBORS | DISCRETE UNIFORM, MIN: 5, MAX: 100 | 5 |
| ALGORITHM | CATEGORICAL: ”auto”, ”ball_tree”, ”kd_tree”, ”brute” | ”ball_tree” |
| WEIGHT | BERNOULLI {uniform, distance} | distance |
| P | BERNOULLI {1, 2} | 1 |

Table S13 **ENET**

| **HYPERPARAMETER** | **SEARCH SPACE** | **FINAL VALUE** |
| --- | --- | --- |
| L1_RATIO | UNIFORM, MIN: 0, MAX: | 0.155 |
| ALPHA | CATEGORICAL, 10^i^ FOR i ∈ {-4, -3, -2, -1, 0, 1} | 10^4^ |

**Inclusion of heart rate in deep-learning models**

Heart rate (HR) and inter-beat intervals (IBI) are not raw sensory modalities recorded with E4 sensors, but features derived from blood volume pressure (BVP) with a proprietary algorithm. HR is given by the E4 as sampled at 1Hz, whereas IBI is an array of values corresponding to the time difference between neighbouring ventricular contractions and, for this reason, it cannot be directly passed through the convolutional encoder of the E4mer, our E4-tailored Transformer architecture. It has been shown in other related fields (e.g.[[1]](#footnote-2),[[2]](#footnote-3)) that artificial neural networks could adaptively learn the most suitable basis functions (i.e. features) for the task at hand without any need to input extracted phenotypes along with raw data. For this reason, we did not consider either HR or IBI in the deep-learning experiments reported in the main manuscript. However, we herewith verified how including HR, along with tri-axial acceleration, BVP, electrodermal activity (EDA), and temperature (EDA), would affect the performance of the fully-supervised E4mer and of the E4mer pre-trained on masked prediction and fine-tuned on the target task. The set of hyperparameters reported above (Tables S3,S7, and S9) were used. In both settings, the inclusion of HR led to a deterioration in performance. In particular, in comparison to ACC_segment_ and ACC_subject_ values of 75.35 and 81.25 for the fully supervised E4mer, inputting HR along with the raw sensory modalities decreased the performance to 74.22 and 79.69 respectively. On the other hand, against ACC_segment_ and ACC_subject_ values of 81.23 and 90.63 for the E4mer pre-trained on masked prediction and fine-tuned on the target task, the inclusion of HR was associated with a drop in performance to 77.76 and 83.76 respectively.

1. Chauhan D, Anyanwu E, Goes J, Besser SA, Anand S, Madduri R *et al.* Comparison of Machine Learning and Deep Learning for View Identification from Cardiac Magnetic Resonance Images. *Clin Imaging* 2022; **82**: 121–126. [↑](#footnote-ref-2)
2. Abrol A, Fu Z, Salman M, Silva R, Du Y, Plis S *et al.* Deep learning encodes robust discriminative neuroimaging representations to outperform standard machine learning. *Nat Commun* 2021; **12**: 353. [↑](#footnote-ref-3)
